# The AP2 Transcription Factor *BrSHINE3* Regulates Wax Accumulation in Nonheading Chinese Cabbage

**DOI:** 10.3390/ijms232113454

**Published:** 2022-11-03

**Authors:** Zhaoyan Huo, Yang Xu, Song Yuan, Jiang Chang, Shuhao Li, Jinwei Wang, Huanhuan Zhao, Ru Xu, Fenglin Zhong

**Affiliations:** College of Horticulture, Fujian Agriculture and Forestry University, Fuzhou 350002, China

**Keywords:** nonheading Chinese cabbage, epidermal waxiness, BSA-seq, preliminary positioning, *BrSHINE3*

## Abstract

Wax is an acellular structural substance attached to the surface of plant tissues. It forms a protective barrier on the epidermis of plants and plays an important role in resisting abiotic and biotic stresses. In this paper, nonheading Chinese cabbage varieties with and without wax powder were observed using scanning electron microscopy, and the surface of waxy plants was covered with a layer of densely arranged waxy crystals, thus differentiating them from the surface of waxless plants. A genetic analysis showed that wax powder formation in nonheading Chinese cabbage was controlled by a pair of dominant genes. A preliminary bulked segregant analysis sequencing (BSA-seq) assay showed that one gene was located at the end of chromosome A09. Within this interval, we identified BraA09000626, encoding an AP2 transcription factor homologous to *Arabidopsis AtSHINE3*, and we named it *BrSHINE3*. By comparing the CDS of the gene in the two parental plants, a 35 bp deletion in the *BrSHINE3* gene of waxless plants resulted in a frameshift mutation. Tissue analysis showed that *BrSHINE3* was expressed at significantly higher levels in waxy plant rosette stage petioles and bolting stage stems than in the tissues of waxless plants. We speculate that this deletion in *BrSHINE3* bases in the waxless material may inhibit wax synthesis. The overexpression of *BrSHINE3* in *Arabidopsis* induced the accumulation of wax on the stem surface, indicating that *BrSHINE3* is a key gene that regulates the formation of wax powder in nonheading Chinese cabbage. The analysis of the subcellular localization showed that *BrSHINE3* is mainly located in the nucleus and chloroplast of tobacco leaves, suggesting that the gene may function as a transcription factor. Subsequent transcriptome analysis of the homology of *BrSHINE3* downstream genes in nonheading Chinese cabbage showed that these genes were downregulated in waxless materials. These findings provide a basis for a better understanding of the nonheading Chinese cabbage epidermal wax synthesis pathway and provide important information for the molecular-assisted breeding of nonheading Chinese cabbage.

## 1. Introduction

Wax is the first barrier that protects plant tissue. It limits the loss of nonstomatal water and helps to prevent damage caused by Ultraviolet (UV), heat, bacterial and fungal pathogens, insects, high salinity, and low temperature [1]. Cuticle wax also plays a role in regulating plant morphology and development through tight binding of the epidermis [2]. Plant epidermal wax is classified as inner epidermal wax or outer epidermal wax. The inner epidermal wax fills the stratum corneum, and the outer epidermal wax assembles into different forms of waxy crystals, including flakes, filaments, rods, tubes, and granular forms, in the outer layer of the stratum corneum [3]. Vegetable wax is a hydrophobic fatty compound extracted from organic solvents that is a complex derivative of very long chain (C20–C36) saturated fatty acids (VLCFAs), usually including alkanes, aldehydes, ketones, primary alcohols, and secondary alcohols [4]. The composition of plant epidermal wax is more complex and varies among plant species. Different organs of the same plant may also show differences, which depend on the developmental stage and environmental conditions [5]. The biosynthetic precursors of epidermal wax are C16 and C18 fatty acids, which are converted into C16 and C18 fatty acyl-coenzyme A (CoA) on the outer plasma membrane of epidermal cells, and these products are transferred to the endoplasmic reticulum [6]. C16 and C18 fatty acyl-CoAs are extended into wax precursors of very low-density lipoproteins with C26 and C34 chains through repeated reactions mediated by the fatty acid elongase (FAE) complex [7]. After extension, long-chain acyl coenzyme A is transformed into wax components through two pathways. The acyl reduction pathway produces primary alcohols and wax esters, and the decarbonization pathway produces alkanes, aldehydes, secondary alcohols, and ketones. Finally, these compounds are transported to the stratum corneum through the ATP binding cassette, ABC transporter, and lipid transporter. In recent years, *Arabidopsis* epidermal wax synthesis-related genes have been widely cloned and studied [8,9,10]. The SHN1/WIN1 AP2 domain protein was the first transcription factor reported to control epidermal wax metabolism. It activates cuticle wax biosynthesis by upregulating the *KCS1*, *CER1*, and *CER2* genes [11]. Subsequent studies have shown that SHN1/WIN1 controls the permeability of the epidermis by regulating the expression of wax biosynthesis genes, especially the long-chain acyl-coenzyme A synthetase LACS2 [12]. The WIN1/SHN1 homologs SHN2 and SHN3 have functions similar to WIN1/SHN1 [13]. In *Arabidopsis*, *AtSHN3* shows activities in addition to the regulation of the stratum corneum metabolic pathway and is involved in mediating the development of floral organs and altering pectin metabolism to modify the epidermal cell wall [14]. The leaves of *Arabidopsis* overexpressing SHN3/WIN3 show a bright phenotype, as waxy crystals are produced on the stem surface, and wax accumulation is increased [15]. CER2 is an acyltransferase involved in the elongation of C28 fatty acids. After *CER2* mutation, studies have shown increases in the levels of C26 and C28 acyl groups, decreases in the levels of primary alcohols and wax lipids, and decreases in the products of the decarboxylation pathway [16]. *CER1* encodes aldehyde decarboxylase, which, together with CER3, catalyzes the formation of oxidized alkanes [17]. In *Arabidopsis*, CER4 encodes a fatty acyl reductase (FAR) that catalyzes the reduction of VLCFA-CoAs to produce primary alcohols in the alcohol formation pathway [7]. Ketoacyl-CoA synthase (KCS) determines the length of different ultralong fatty acid chains, and the enzyme shows fatty acid substrate specificity. For example, KCS1 is involved in the extension of C22-24 long-chain fatty acid chains, and KCS2 is involved in the extension of C20-22 long-chain fatty acids [10].

Waxy genes have been cloned in many plants, such as tomato [18], apple [19], rice [8], and corn [20]. The waxy surfaces of the leaves and stems of *Brassica* plants of the cruciferous family, such as *Brassica oleracea* L. *var.capitata* L., cabbage, *Brassica rapa* var. chinensis ‘Parachinensis’, and *Brassica pekinensis* Rupr., usually have a frosty white appearance. Recently, the epidermal wax synthesis regulatory gene *Br013809* has been located in Chinese cabbage, and its homologous gene in *Arabidopsis* is *brwax1* [21]. The *BoGL1* gene, which controls the bright phenotype, has been finely mapped in cabbage, and its homologous gene is *CER1* [22]. Loss of function of the *brcer1* gene in nonheading Chinese cabbage leads to a waxless phenotype [17]. In *Porphyra* stalk, the candidate waxy genes *Bra011487* and *Bra011470* were located on chromosome A01 through the genetic linkage of traits [23]. *Brassica rapa* L. ssp. *chinensis* L. is widely grown in Southeast Asia and Europe. The waxy epidermis on its leaves and stems provides a protective film to limit water loss and protect plants from abiotic stress [24]. Based on previous studies [25], we analyzed the differences in morphology and physiological indexes of epidermal cells of waxy and waxless materials under high-temperature stress, and we revealed the physiological mechanism of waxy in nonheading cabbage in response to high-temperature stress. Second, based on the phenotype identification and genetic analysis of wax, a gene homologous to one involved in wax synthesis in *Arabidopsis thaliana* was identified by bulked segregation analysis (BSA) and named *BrSHINE3*; this gene was further cloned and analyzed. Then, combined with the transcriptome data, the downstream differentially expressed genes of *BrSHINE3* were mined for homologous cloning, and their gene expression levels in different growth stages and under high-temperature stress were analyzed. To clarify the regulation and molecular mechanism of waxy biosynthesis in nonheading cabbage. The aim of this study was to reveal the mechanism of wax metabolism in nonheading cabbage at the physiological and molecular levels and to provide a theoretical basis for further study of wax synthesis in Brassica crops.

## 2. Results

### 2.1. Wax Phenotype Identification and Genetic Analysis

**Figure 1:**
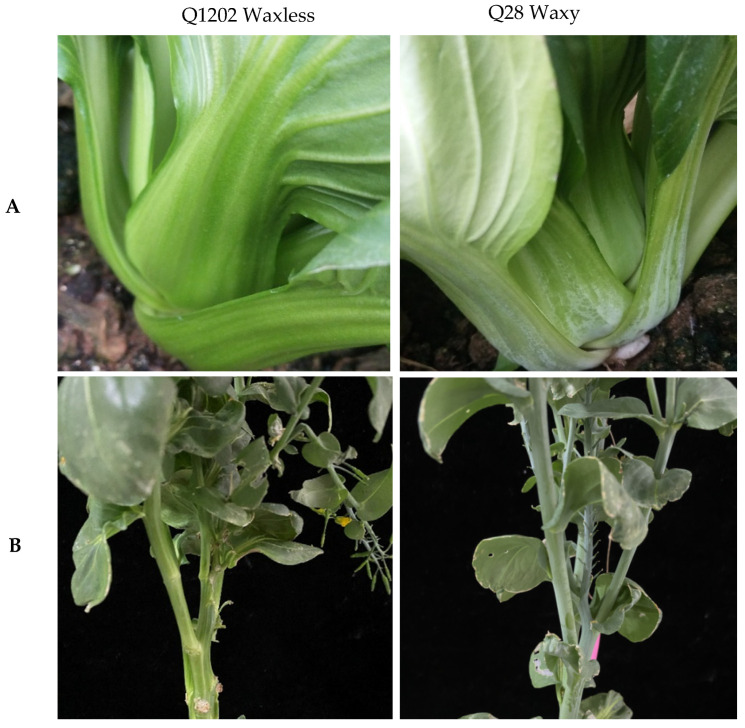
Phenotypes of Q1202 and Q28 at rosette (**A**) and shoot stages(**B**).

**Figure 2:**
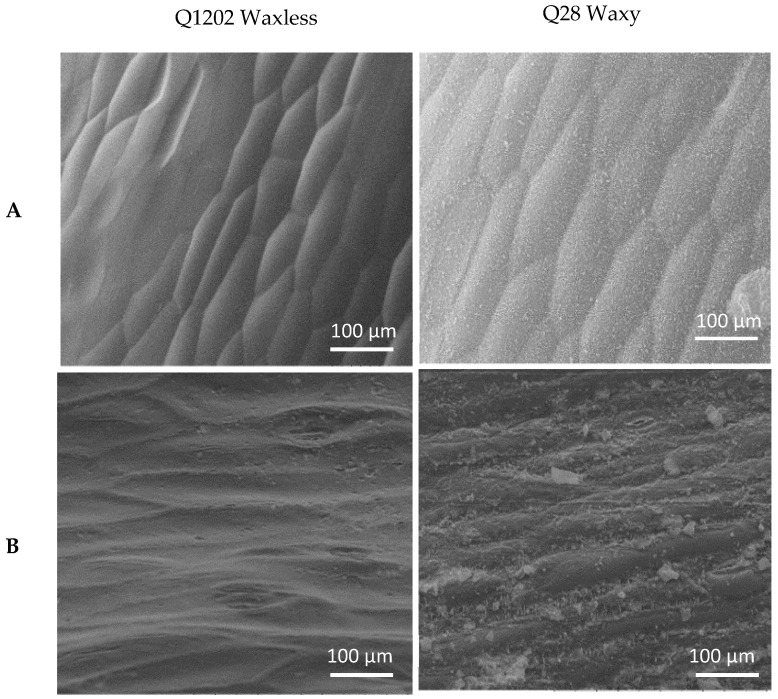
Scanning electron micrographs of Q1202 and Q28 at the rosette (**A**) and shoot stages (**B**).

The petioles and stems of Q28 were covered with white powdery wax, while the petioles and stems of Q1202 were smooth and green without wax (Figure 1). SEM showed that the surface of Q28 plants was covered with closely arranged waxy crystals, while the density of waxy crystals on the surface of Q1202 plants was significantly lower, with almost no waxy crystals observed (Figure 2). Regardless of whether the parents Q1202 and Q28 were orthogonal or hybrid, their offspring showed the same wax phenotype as Q28, and no F1 individuals without wax were observed, indicating that a nuclear gene encoding the wax trait on the surface of nonheading cabbage plants existed. The segregation of waxy and nonwaxy traits occurred in the F2 population. The separation ratio was 2.88:1, and the chi-square test conformed to the theoretical value of 3:1, indicating that the surface of nonheading cabbage was waxy. These quality-related traits are controlled by a pair of genes, and backcross experiments were conducted between F1 plants and the parents to strengthen the performance of parental traits and confirm the results (Table 1).

### 2.2. Location and Sequence Analysis of the BrSHINE3 Gene

**Figure 3:**
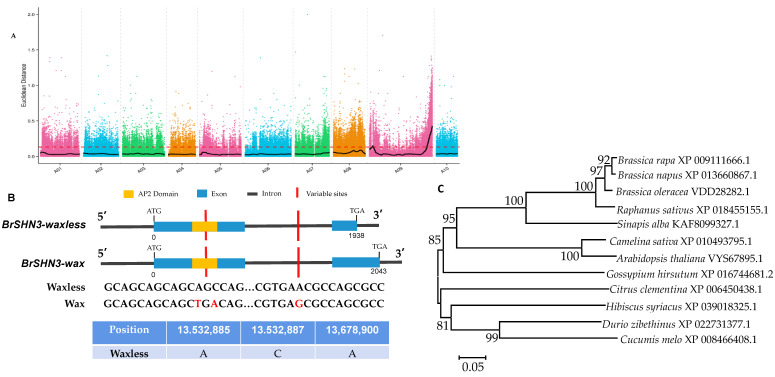
Distribution of ED on chromosomes between interpools (**A**), analysis of waxy candidate gene mutation sites (**B**), and phylogenetic tree of *BrSHINE3* of each species (**C**).

BSA-seq technology was used to locate candidate genes required for wax synthesis. By considering overlapping regions, the SNP and indel association analysis indicated a candidate region with a length of 0.8 Mb on chromosome 9 (2,860,000–3,660,000 bp) containing 153 genes (Figure 3A). All 153 genes in the candidate region were functionally annotated, among which 35 nonsynonymous mutant genes were identified between the parents, and 9 frameshift mutant genes were annotated. As *BraA09000626* is highly homologous to *SHINE3* (AT5G25390), which has been confirmed to be involved in wax biosynthesis in *Arabidopsis*, we conducted a detailed analysis of this gene. We amplified the full-length cDNAs from plants with the waxy and waxless phenotypes. Sequence alignment showed a 35 bp deletion of the *BrSHINE3* gene resulting in a frameshift mutation in the waxless phenotype plants, and the corresponding amino acid sequence was also missing (Figure 3B). Therefore, we speculated that this deletion in the waxless varieties affects the function of the *BrSHINE3* gene in wax synthesis by inhibiting the biosynthesis of wax, thus resulting in a change in wax deposition between varieties. At the same time, the amino acid sequence of *BrSHINE3* was compared with that of *Brassica napus*, *Raphanus sativus*, *Brassica oleracea*, *Arabidopsis thaliana*, *Sinapis alba*, *Camelina sativa*, *Gossypium hirsutum*, *Citrus clementina*, *Hibiscus syriacus*, *Durio zibethinus*, *Cucumis melo*, and other species, and cluster analysis was performed to construct the phylogenetic tree (Figure 3C). The results showed that *SHINE3* in nonheading cabbage was more closely related to genes in *Brassica napus*, *Raphanus sativus*, and *Brassica oleracea*.

### 2.3. Analysis of the Subcellular Localization of the BrSHIN3 Gene

**Figure 4:**
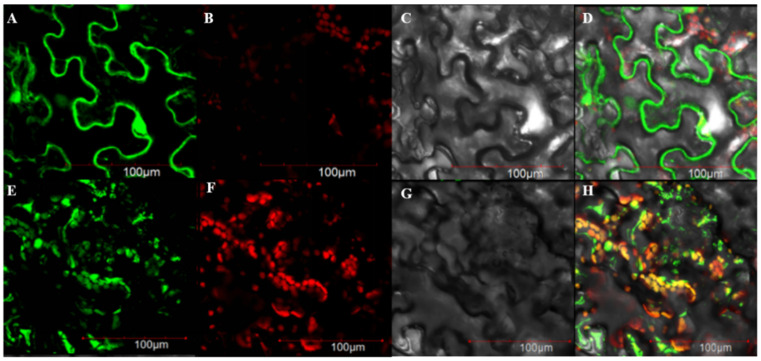
Transient expression of *BrSHINE3* in tobacco epidermal cells. (**A**,**E**) GFP is fluorescence field; (**B**,**F**) is chloroplast channel; (**C**,**G**) is open field; (**D**,**H**) is Merge, which means superposition; (**A**–**D**) is 35S-GFP; (**E**–**H**) is 35S:*BrSHINE3*-GFP.

BrSHN3 was predicted to be localized in the chloroplasts and nucleus. Tobacco leaves were observed 48 h after injection under a confocal microscope (Figure 4). 35S-GFP was detected in the fluorescence field, and the superimposed field showed a green fluorescence signal in the nucleus and cell membrane. 35S:*BrSHINE3*-GFP was detected in the fluorescence field and superimposed field, and the green fluorescence signal was found in the nucleus of the superimposed field. In the superimposed field, the green fluorescence signal appeared in the nucleus of brown algae. The overlapping positions of green and red fluorescence signals in the superimposed field were concentrated in chloroplasts (Figure 4E–G), and the results were consistent with the predicted results. These results indicate that the 5′-terminus of the protein functions as a transport peptide, and the protein encoded by this gene is localized to chloroplasts. Thus, *BrSHINE3* is localized in the nucleus and chloroplast, and we speculate that this gene may function as a transcription factor.

### 2.4. Tissue Expression Analysis and Functional Verification of the BrSHINE3 Gene

**Figure 5:**
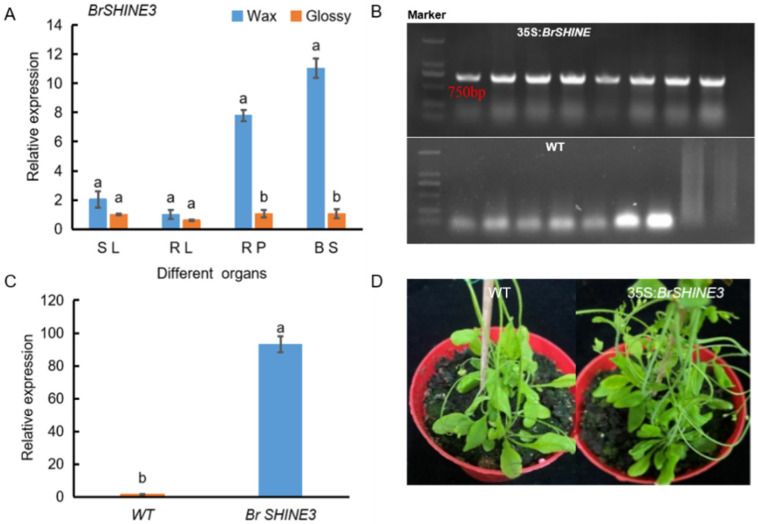
The expression profile of candidate gene BrSHINE3 was analyzed (**A**), molecular verification of transgenic Arabidopsis (**B**), expression level analysis of transgenic Arabidopsis (**C**) and phenotype analysis of transgenic plants (**D**). Different lowercase letters in the same column indicate significant differences at the 0.05 level among treatments.

The expression pattern of the *BrSHINE3* gene in nonheading Chinese cabbage was analyzed using fluorescence-based quantification technology. The expression level of the *BrSHINE3* gene was different in four different parts of waxy nonheading Chinese cabbage (seedling leaves, rosette leaves, rosette petioles, and moss petioles). In waxy nonheading Chinese cabbage, the expression level was higher in the petiole and stem at the lotus root stage but was lower in nonwaxy plants (Figure 5A). The constructed overexpression vector p1302:*BrSHINE3* was used to infect wild-type *Arabidopsis thaliana* via the *Agrobacterium*-mediated method. After *A. thaliana* matured, the T0 generation seeds were collected, and the T1 generation resistant lines were screened on 1/2 MS solid plate medium containing 25 mg/L hygromycin. Ten T1 generation-resistant plants were obtained. The *Arabidopsis* T1 generation seedlings were transplanted onto the substrate and cultured under the following conditions: relative humidity of 70%, temperature of 23 °C, light intensity of 100 μmol m^−2^s^−1^, and light–dark cycle of 14 h/8 h. When the *Arabidopsis* T1 seedlings reached 1 month old, leaf DNA was extracted, and PCR was performed to identify the positive plants. Eight independent resistant plants were selected, and RT–PCR detection was performed using the upstream vector P1302 and downstream primers corresponding to the target gene. The target bands were detected in the eight selected T1 transgenic *Arabidopsis* lines, indicating that the *BrSHINE3* gene had been successfully transferred into *Arabidopsis* (Figure 5B). Fluorescence quantitative PCR was used to detect the expression level of the *BrSHINE3* gene in transgenic *Arabidopsis*. As shown in Figure 5C, the relative expression levels of *BrSHINE3* were high in transgenic plants and extremely low in wild-type Arabidopsis. A phenotypic analysis of transgenic *Arabidopsis* showed that, in the same growth period, the stem surface of wild-type *Arabidopsis* was smooth green, and the stems of *A. thaliana* overexpressing *BrSHINE3* were covered with wax, consistent with previous findings (Figure 5D); therefore, the *BrSHINE3* gene was involved in wax metabolism in nonheading Chinese cabbage.

### 2.5. Transcriptome and Differential Gene Pathway Analyses

**Figure 6:**
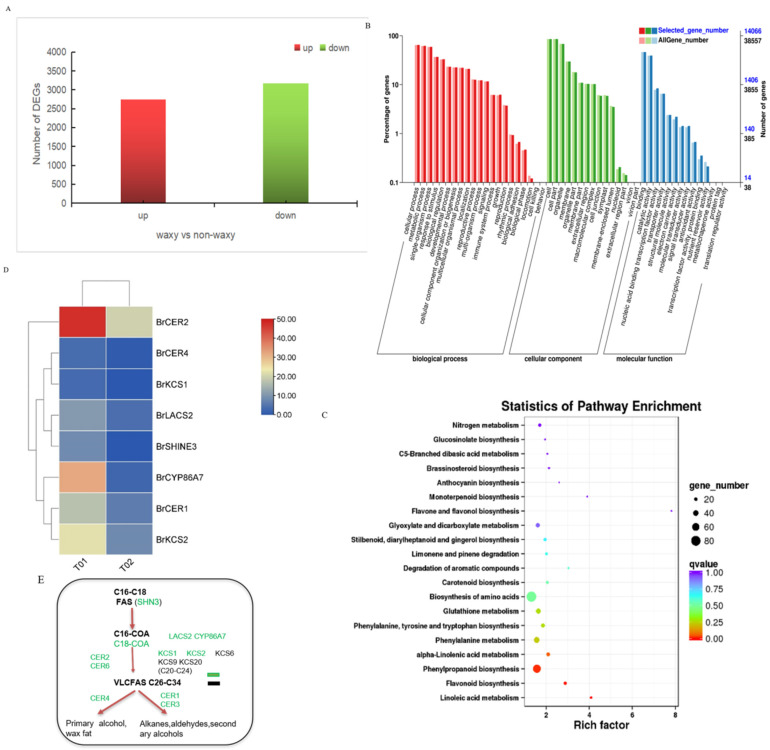
Transcriptome and differential gene pathway analysis.Number of DEGs (**A**), KEGG classification (**B**), KEGG enrichment map (**C**), Analysis of differential gene expression changes (**D**), Wax metabolic pathway (**E**).

By transcriptome analysis, 5907 DEGs were identified between waxy and waxless nonheading cabbage, of which 2737 were upregulated and 3170 were downregulated (Figure 6A), and multiple metabolic pathways were screened (see Supplementary Data S4). Among them, we focused on several pathways related to wax metabolism, such as fatty acid elongation (ko00062, 8), fatty acid degradation (ko00071, 14), fatty acid metabolism (ko01212, 15), fatty acid biosynthesis (ko00061, 12), and saturated fatty acid biosynthesis (ko01040, 10) (Figure 6C). As shown in Figure 6D, seven differentially expressed genes in the fatty acid metabolism pathway, *BraA010016217*, *BraA03006420*, *BraA04002725*, *BraA05002026*, *BraA06002725*, *Bra_newGene3249.1*, and *Bra_newGene2589.1*, were found to be significantly upregulated in waxy materials. Based on a sequence comparison, these genes were homologous to the wax synthesis genes *CER2*(AT4G24510), *CYP86A7*(At1G63710), *CER1*(At1G02205), *LACS2*(At1G49430), *CER4*(At4G33790), *KCS1*(AT1G01120), and *KCS2*(At1G04220) reported in *Arabidopsis*, and they were named *BrCER2*, *BrCYP86A7*, *BrKCS2*, *BrLACS2*, *BrCER4*, *BrKCS1*, and *BrCER1*, respectively. Studies have shown that these genes are involved in different pathways regulating wax synthesis-related products. Among these genes, *CER2* is involved in the biosynthetic pathway from acetyl-CoA to ultralong fatty acid chains, *CER1* is involved in the decarboxylation pathway, *CER4* is involved in the acyl reduction pathway, and *KCS1* and *KCS2* are involved in the biosynthetic pathway from acetyl-CoA to C20-C24 fatty acid chains [26,27]. LACS2 CYP86A7 is involved in the acetyl-CoA biosynthetic pathway (Figure 6E).

### 2.6. qPCR Verification of Downstream Genes

**Figure 7:**
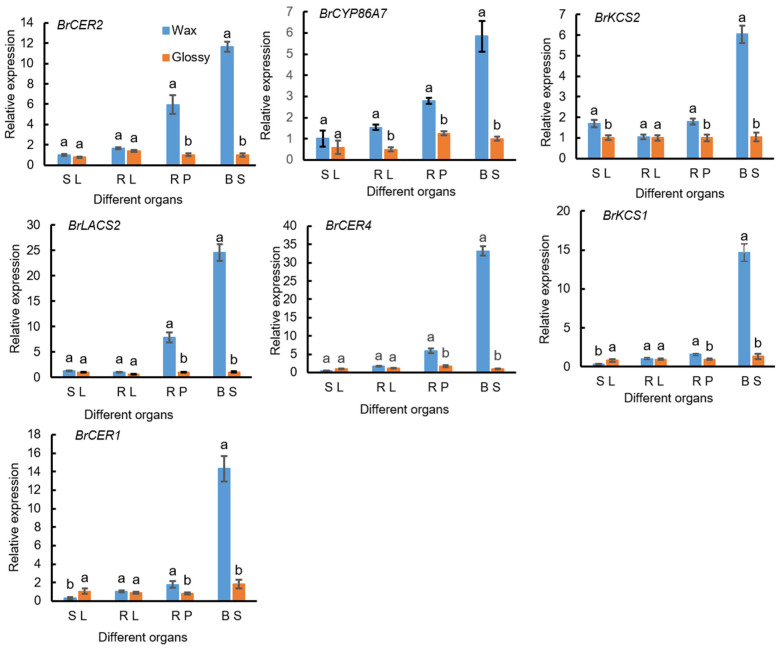
Expression analysis of thelabyte differentially synthesized genes in thelabyte leaf (SL), Rosette leaf (RL), Rosette petiole (RP) and Bolting stem (BS) tissues. Data are presented as the mean ± SD of three independent biological replicates. Different lowercase letters in the same column indicate significant differences at the 0.05 level among treatments.

**Figure 8:**
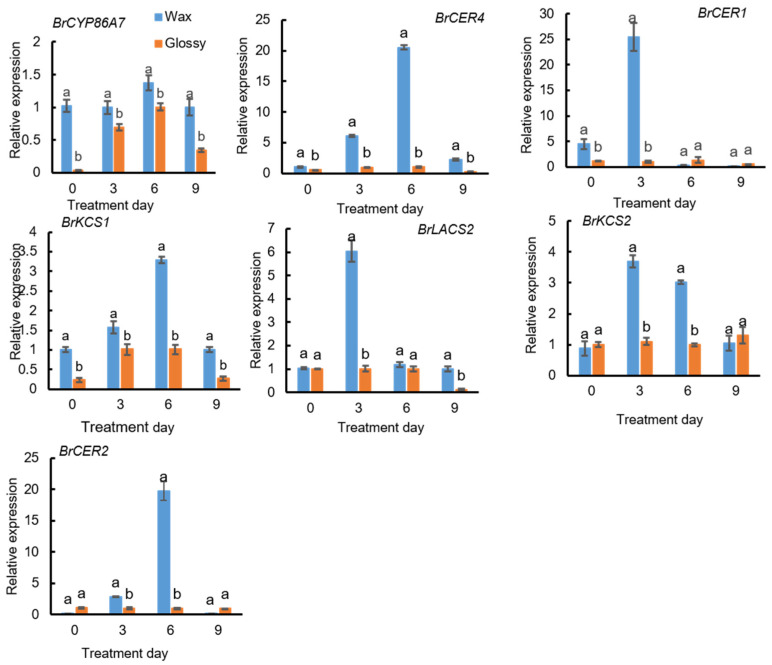
Expression analysis of waxy genes under high-temperature stress. Data are presented as the mean ± SD of three independent biological replicates. Different lowercase letters in the same column indicate significant differences at the 0.05 level among treatments.

The expression levels of seven DEGs, *BrCER2*, *BrCYP86A7*, *BrKCS2*, *BrLACS2*, *BrCER4*, *BrKCS1*, and *BrCER1*, were detected using qPCR in different parts of waxy and waxless plants and plants under high-temperature stress. Significant differences in the expression levels of DEGs were observed in different tissues of waxy plants, among which higher expression was detected in the petioles at the rosette stage and the flowering stems at the bolting stage. The expression levels of these seven genes were relatively low in waxless phenotype plants (Figure 7). After exposure to high-temperature stress for 3 and 6 d, the seven DEGs showed higher expression in waxy phenotype materials than in waxless phenotype materials, and the differences were significant. With the prolongation of high-temperature stress, the expression of the eight genes showed dynamic changes, first increasing and then decreasing. Among these genes, the expression levels of *BrKCS2*, *BrLACS2*, and *BrCER1* in waxy plants reached a maximum after 3 d of high-temperature stress, and the expression levels of *BrCER2*, *BrCYP86A7*, *BrCER4*, and *BrKCS1* reached a maximum after 6 d of high-temperature stress (Figure 8); therefore, the expression of key genes involved in wax synthesis in nonheading Chinese cabbage exhibits spatial and temporal differences and is regulated by temperature.

## 3. Discussion

Epidermal wax is a kind of noncellular structural material attached to plant epidermal tissue. As the first protective barrier of plant tissue, epidermal wax plays an active role in resisting environmental stress and ensuring normal plant growth and development [28]. Studies have shown that the accumulation of wax in *Brassica napus* leaves is conducive to maintaining water balance in the plant [29]. The wax coating on the leaf surface of cabbage can effectively inhibit the invasion of pests [30]. The accumulation of wax in *Arabidopsis thaliana* can regulate the permeability of epidermal cells and enhance the heat tolerance of plants [31]. Many studies have found that the waxy surfaces of the leaves and stems of cruciferous plants usually appear to be covered with glaucous frost. The analysis of the waxy phenotype of nonheading Chinese cabbage revealed that the petioles and stems were covered with hoarfrost-like wax. Under the SEM, the wax appears as scales and rods. In cabbage, the lack of waxiness is controlled by a dominant gene [32]. Zhu et al. studied the waxless traits of cabbage rape and Indian mustard and found that their waxless traits are controlled by a pair of recessive genes [33]. The waxy trait of common cabbage is the same as those of cabbage, cabbage stalk, rape, and Chinese cabbage, all of which present dominant inheritance and are controlled by a pair of dominant genes [34]. As shown in the present study, the waxy trait of nonheading Chinese cabbage is controlled by a pair of nuclear genes that exhibit dominant inheritance. In a previous study, Liu et al. used SSR markers to successfully clone candidate genes controlling the waxy cabbage mutants 10Q-961 and g21-3 and identified the *CGL1* gene, which is a homolog of *CER1* related to wax synthesis in *Arabidopsis* and is related to wax synthesis. The candidate gene *brcer1* of nonheading cabbage (*B. rapa* L.) was isolated by performing a homology analysis and identified as a homologous gene of *CER1* [35]. In our study, preliminary mapping of the waxy gene was performed based on genetic analysis. A gene homologous to *SHINE3* was identified by conducting a nucleotide analysis. A 39 bp deletion detected in the third exon of the *brcer4* gene in the smooth mutant of *Porphyra stipa* resulted in a premature stop codon in the transcript of the smooth mutant [36]. In the present study, a 35 bp deletion in the exon region of the *BrSHINE3* gene in smooth plants resulted in aberrant mRNA transcription.

The AP2/ERF transcription factor WIN1/SHN1 and its homolog SHN2 and SHN3 are reported to activate epidermal wax biosynthesis [37]. More recent studies have shown that WIN1/SHN1 directly regulates the biosynthesis of the stratum corneum and indirectly affects the production of stratum corneum wax. The protein sequence homology of SHN1/WIN1 and SHN2 was 55%, and the homology of SHN2 and SHN3 was higher [38]. The *Arabidopsis SHN2* gene is mainly expressed in anther and silique tissues, and the *SHN3* gene is expressed in all plant organs. The WIN/SHN1 homologous gene in apple showed low expression in the peel and leaves [39]. An analysis of gene expression patterns showed that *BrSHINE3* presented different expression levels in various parts of nonheading Chinese cabbage at different stages, and the highest expression level was detected in the flowering stems at the bolting stage and the petioles at the mature stage. According to some studies, *SHIN1* overexpression leads to a higher wax content in transgenic tomatoes compared to the wild-type strain, along with higher expression of some genes related to wax synthesis [40]. In *Arabidopsis*, overexpression of the other two members of the *SHN* gene family, *SHN2* and *SHIN3*, produces a phenotype that is very similar to that of WIN1/SHN1 transgenic plants. The overexpression of *AtSHIN* alters epidermal characteristics and increases the waxiness of the *Arabidopsis* epidermis [41]. The present study showed that when the *BrSHINE3* gene was overexpressed in *Arabidopsis*, the leaf surface was shinier than that of the wild-type strain, and wax accumulation occurred in the stem, consistent with the results from a study by Marsch-Martinez et al. on the *Arabidopsis SHIN3* mutant [42]. In plants, leaf surface wax synthesis mainly occurs in the epidermal cells of leaves. The precursors of fatty acids in wax synthesis are mainly C16 and C18 fatty acids, which are mainly synthesized in the chloroplasts of plant epidermal cells. According to previous studies [43], the process of plant wax synthesis is very clear; the synthesis of fatty acids is initially carried out in the chloroplasts of plants. More importantly, the subcellular localization of the BrSHIN3 protein in chloroplasts showed that the BrSHIN3 protein may participate in or affect chloroplast synthesis or degradation pathways, leading to the formation of wax. This subcellular localization is different from most of the previously reported subcellular localization results of wax synthesis-related proteins, which are mostly in the endoplasmic reticulum [44,45,46]. However, the observed localization to chloroplasts may be due to the limitations of experimental conditions and equipment and may be influenced by chloroplast autofluorescence. In tomato, SHN3 may also be indirectly regulated via the HD-ZIP or MIXTA protein. The target genes downstream of *SlSHN3* that encode members of the CYP86A subgroup are critical for the formation of wax on the surface of tomato fruits by acting directly on the *SlSHN3* promoter to control the metabolism of the epidermal stratum corneum and cell wall [47]. Using genetic methods, many genes related to wax regulation have been identified in *Arabidopsis* and other crops, including *CER1*, *KCS1*, *PAS2*, *CER2*, *CER4*, *WSD1*, *LACS2*, *ABCG11*, *WBC11*, *MYB96*, and *LTPG1*. The AP2-ERF transcription factor also inhibits cuticle wax biosynthesis by upregulating the *KCS1*, *CER1*, and *CER2* genes [11]. Studies on WIN1/SHIN1 have shown that it directly controls the expression of the *LACS2*, *GPAT4*, *CYP86A4*, *CYP86A7*, and *HTH* genes involved in wax biosynthesis, and it only indirectly regulates stratum corneum wax deposition [11]. In this study, seven genes, *BrCER2*, *BrCYP86A7*, *BrKCS2*, *BrLACS2*, *BrCER4*, *BrKCS1*, and *BrCER1*, were identified in combination with the transcriptome, and their expression levels in waxy plants were higher than those in nonwaxy plants.

In conclusion, we identified the *AtSHINE3* homolog *SHINE3*, which plays an important role in the wax synthesis in cabbage. In addition, subcellular localization and related functional analyses were carried out, which confirmed that the *BrSHINE3* gene was involved in wax metabolism in nonheading Chinese cabbage. Overexpression analysis of *BrSHINE3* in *Arabidopsis* showed that the relative expression level in transgenic plants was approximately 90 times higher than that in wild-type Arabidopsis. The phenotype of transgenic *Arabidopsis* in the same growth period shows that the stem surface of wild-type *Arabidopsis* was smooth and green, and the stem of Arabidopsis overexpressing *BrSHINE3* was covered with wax, consistent with previous findings. Further studies on the function of *BrSHINE3* will provide more information to elucidate its role in wax biosynthesis. These studies will provide a new method for gene cloning and molecular breeding.

## 4. Materials and Methods

Two nonheading Chinese cabbage inbred lines, Q1202 and Q28, were used as parental strains to construct a segregating population. Q1202 has a waxless phenotype, and Q28 has a waxy phenotype. The phenotype basically remains the same from the plant harvest stage to the seed maturity stage, which is easy to observe. Q1202 and Q28 were used to construct F1, F2, BC1, and BC2 populations for genetic and localization studies. The F1 generation was backcrossed with Q1202 and Q28 to obtain the BC1 and BC2 populations, and the same plant was used as the female parent in both backcrosses. All plant materials were provided by the Facility Vegetable Seed Industry Innovation Group of Fujian Agriculture and Forestry University and were planted at the Baisha Experimental Base of Minhou, Fujian Province. All plants in the rosette stage and bolting stage were observed, and the waxy properties of the epidermis were visually inspected. The separation rate of the F2 and BC1 populations was determined with the χ^2^ test.

### 4.1. Scanning Electron Microscopy (SEM) Analysis

SEM was used to study the leaf surfaces of Q1202 and Q28 plants. Petioles or stems of waxy nonheading Chinese cabbage were analyzed during rosette and bolting stages, and each sample of leaves was cut into small pieces (approximately 5 mm^2^) with a razor blade. The leaf pieces were then adhered to the sample table with conductive double-sided adhesive and placed under a vacuum pump. The air was extracted, and the phenotype was analyzed under an SEM (TM3030 PLUS, HITACHI, Tokyo, Japan).

### 4.2. Preliminary Localization of Candidate Genes

In total, 50 individual waxy plants and 50 individual waxless plants were selected from the parental and F2 populations, respectively, and new upper leaves were collected, placed in liquid nitrogen, and stored at −80 °C. Total DNA was extracted using the hexadecyltrimethylammonium bromide (CTAB) method, and its concentration was measured with a NanoDrop 2000 spectrophotometer (Invitrogen Fisher, Beijing, China). The DNA was then uniformly diluted to 50 ng L^−1^ and stored at −80 °C until use. The DNA of 50 individual waxy plants and the DNA of 50 wax-containing plants, and 50 waxless plants were mixed equally to construct a waxless mixed pool and a wax-free mixed pool. The two pooled DNA samples and two parental DNA samples were processed according to standard procedures for sample detection, library construction, library quality detection, and computer sequencing provided by Illumina (Biomark Biotechnology Co., Ltd., Beijing, China). Please refer to the Chinese Cabbage Genome Database (http://www.genoscope.cns.fr/brassicanapus/data/ (accessed on 11 May 2020)). The raw reads obtained from sequencing were subjected to the removal of linkers and low-quality sequences (reads), and high-quality sequence data (clean reads) were obtained for the subsequent informatics analysis. GATK software was used to detect single nucleotide polymorphisms (SNPs) and indels as labels of multiple samples, Picard was used for deduplication, and SnpEff software was used to annotate SNPs and indels (for details, see Supplementary Data S1).

### 4.3. DNA Extraction and Polymerase Chain Reaction (PCR) Amplification

The CTAB method was used to extract genomic DNA from the fresh leaves of the parents and F2 individuals, and the concentration of each genomic DNA sample was determined using a spectrophotometer and adjusted to 50 ng/µL (for details, see Supplementary Data S2).

### 4.4. Preparation of the Plant Fusion Expression Vector and Production of Transgenic Arabidopsis

For the construction of the P1302-35S-BrSHN3-GFP vector, a *BrSHINE3* gene fragment was amplified from waxy plant leaf cDNA using the specific primers F: ggactcttgaccatggatgaacactactaaagacctttctcaaaaag and R: gtcagatctaccatggatgactaagacctgaaacggc. The plant binary expression vector pcambia1302 was digested with NcoI and purified. The pcambia1302 vector contains a 35S enhancer and an MfGFP vector tag. The purified PCR product was ligated to the digested vector by homologous recombination to obtain the recombinant plasmid pcambia1302-BrSHN3 (for details, see Supplementary Data S3).

### 4.5. Subcellular Localization of BrSHINE3

The fusion expression vector 35S:*BrSHINE3*-GFP was transformed into competent *Agrobacterium* GV3101 cells, and the cells were evenly spread on Luria-Bertani solid culture medium containing 50 mg·L^−1^ kanamycin sulfate and 100 mg·L^−1^ rifampicin antibiotics, inverted, and placed in a 28 °C incubator. After 48 h of culture, a single colony was picked and grown in a liquid medium with shaking for PCR detection. The positive clones were cultured in a Luria-Bertani liquid medium containing 15 mL of 50 mg·L^−1^ kanamycin and 100 mg·L^−1^ rifampicin antibiotics at 28 °C and 180 r·min^−1^ for 24 h. When the OD_600_ of the bacterial solution reached 0.8, the bacteria were collected by centrifugation at 5000 r·min^−1^ for 3 min and resuspended in 10 mmol·L^−1^ MgCl_2_, 10 mmol·L^−1^ MES-KOH (pH 5.6), and 100 μmol·L^−1^ acetosyringone at a final concentration corresponding to an OD_600_ of 0.8. After incubation at room temperature for 2–4 h, a 1 mL sterile syringe was used to aspirate the bacterial solution and inject it into the tobacco leaves, taking care to avoid the veins. After the infected tobacco plants were cultured for 3 d under 16 h/8 h light/dark conditions with day and night temperatures of 22 °C and 18 °C, respectively, the lower surface of the leaf tissues was removed and observed under a laser confocal microscope to analyze the subcellular localization of the BrSHINE3 protein.

### 4.6. Transcriptome Analysis

The transcriptome data of the test samples were generated by Biomark (Beijing, China). The Illumina high-throughput sequencing platform was used to sequence the cDNA library, the data were filtered to obtain clean data, and the sequence was compared with the reference genome of Chinese cabbage (*Brassica rapa pekinensis*). Cufflinks software was used to quantitatively detect the FPKM expression level of the genes being compared, and a fold change (fold difference) of 2 and FDR (false discovery rate) < 0.01 were used as the screening criteria. GO (Gene Ontology Resources, http://geneontology.org/ (accessed on 11 May 2020)), KEGG (Kyoto Encyclopedia of Genes and Genomes, https://www.kegg.jp/ (accessed on 11 May 2020)), and COG (Genome Clusters, https://www.kegg.jp/ (accessed on 11 May 2020)) tests were performed using BLAST software. Homologous protein group, https://www.ncbi.nlm.nih.gov/research/cog-project/ (accessed on 11 May 2020)) and Pfam (http://pfam.xfam.org/ (accessed on 11 May 2020)) databases were used for functional annotation and enrichment analyses.

### 4.7. Gene Expression Analysis

TRIzol reagent (Invitrogen Fisher, Beijing, China) was used to extract total RNA from different tissues, and the TaKaRa PrimeScript^TM^ RT Reagent Kit with gDNA Eraser (Perfect Real Time (accessed on 11 October 2021)) was used to generate cDNA templates from 1 µg of total RNA according to the manufacturer’s instructions (for details, see Supplementary Data S4).

### 4.8. Statistical Analyses

Statistical analyses were performed using DPS 17.10 software (Zhejiang University, Hangzhou, China). Duncan’s multivariate difference test (α = 0.05) was used to analyze the significance of differences among the different treatments.

## 5. Conclusions

In this study, waxy (Q28) and waxless (Q1202) varieties of Chinese cabbage were used as experimental materials to analyze the physiological response mechanism of epidermal wax to high-temperature stress. On the basis of waxy phenotype identification and genetic characteristics analysis, a separate population was constructed from the parents of nonheading cabbage and their subsequent generations. A homologous gene involved in waxy synthesis in *Arabidopsis thaliana* was located by BSA technology and named *BrSHINE3*, which was further cloned and analyzed. To clarify the mechanism of wax metabolism in Chinese cabbage without head formation and provide a theoretical basis for the further study of wax synthesis in Brassica crops, homologous cloning was carried out in combination with transcriptome analysis and gene expression levels in different growth stages and under high-temperature stress were analyzed.

## Figures and Tables

**Table 1:** Genetic analysis of waxy characters in parents and generations of nonheading Chinese cabbage.

Generations	Waxy	Glossy	Separation Ratio	Expectations Ratio	χ^2^
P1 (Q28)	41				
P2 (Q1202)	0	52			
F1 (P1 × P2)	163	0			
F1 (P2 × P1)	179	0			
BC1 (F1 × Q28)	157	0			
BC1 (F1 × Q1202)	64	81	0.79:1	1:1	1.45
F2	168	58	2.88:1	3:1	1.76

## Data Availability

The data presented in this study are available on request from the corresponding author.

## Supplementary Materials

The following supporting information can be downloaded at: https://www.mdpi.com/article/10.3390/ijms232113454/s1.
