# Supplementary material for: The AP2 Transcription Factor BrSHINE3 Regulates Wax Accumulation in Nonheading Chinese Cabbage
Source: Int J Mol Sci. 2022 Nov 3;23(21):13454. doi: 10.3390/ijms232113454 (PMC9656708; doi:10.3390/ijms232113454)
Supplement: Supplementary file 1 [file ijms-23-13454-s001.zip › ijms-1959036-supplementary.pdf]

Title: The AP2 Transcription Factor *BrSHINE3* Regulates Wax Accumulation in Nonheading Chinese Cabbage

Zhaoyan Huo, Yang Xu, Song Yuan, Jiang Chang, Shuhao Li, Jinwei Wang, Huanhuan Zhao, Ru Xu and Fenglin Zhong

Supplementary Data S1

4.2. Preliminary localization of candidate genes

Wax

mnttkdlsqkekkstrkmfymflwsvtytvifiwcvfflfniylsthqhhkyyfvsyliirvcvirkrrvwlgtfntaeeaarvydq
aavlmngqnaktnfpviksngsvspdvntplsellnaklrknckdqtpyltclrldnnsshigvwqkragssssqnwvklvelgdgvsa
sagdtgtnklkkgnddveeedqnsmqmieellnwtcpasasvsgl

ATGAACACCACCAAGGACCTGAGCCAGAAGGAGAAGAAGAGCACCAGGAAGAT
GTTCTACATGTTCCTGTGGAGCGTGACCTACACCGTGATCTTCATCTGGTGCGTGTTCT
TCCTGTTCAACATCTACCTGAGCACCCACCAGCACCACAAGTACTACTTCGTGAGCTAC
CTGATCATCAGGGTGTGCGTGATCAGGAAGAGGAGGGTGTGGCTGGGCACCTTCAAC
ACCGCCGAGGAGGCCGCCAGGGTGTACGACCAGGCCGCCGTGCTGATGAACGGCCAG
AACGCCAAGACCAACTTCCCCGTGATCAAGAGCAACGGCAGCGTGAGCCCCGACGTG
AACACCCCCCTGAGCGAGCTGCTGAACGCCAAGCTGAGGAAGAACTGCAAGGACCA
GACCCCCTACCTGACCTGCCTGAGGCTGGACAACAACAGCAGCCACATCGGCGTGTG
GCAGAAGAGGGCCGGCAGCAGCAGCAGCTGACAGAACTGGGTGAAGCTGGTGGAGC
TGGGCGACGGCGTGAGCGCCAGCGCCGGCGACACCGGCACCAACAAGCTGAAGAAG
GGCAACGACGACGTGGAGGAGGAGGACCAGAACAGCATGCAGATGATCGAGGAGCT
GCTGAACTGGACCTGCCCCGCCAGCGCCAGCGTGAGCGGCCTG

Waxless

Mnttkdlsqkekkstrkmfymflwsvtytvifiwcvfflfniylsthqhhkyyfvsyliirvcvirkrrvwlgtfntaeeaarvydq
aavlmngqnaktnfpviksngsvspdvntplsellnaklrknckdqtpyltclrldnnsshigvwqkragsssssqnwvklvelgdgvn
asagdtgtnklkkgnddveeedqnsmqmieelltdmp

ATGAACACCACCAAGGACCTGAGCCAGAAGGAGAAGAAGAGCACCAGGAAGAT
GTTCTACATGTTCCTGTGGAGCGTGACCTACACCGTGATCTTCATCTGGTGCGTGTTCT
TCCTGTTCAACATCTACCTGAGCACCCACCAGCACCACAAGTACTACTTCGTGAGCTAC

CTGATCATCAGGGTGTGCGTGATCAGGAAGAGGAGGGTGTGGCTGGGCACCTTCAAC

ACCGCCGAGGAGGCCGCCAGGGTGTACGACCAGGCCGCCGTGCTGATGAACGGCCAG

AACGCCAAGACCAACTTCCCCGTGATCAAGAGCAACGGCAGCGTGAGCCCCGACGTG

AACACCCCCCTGAGCGAGCTGCTGAACGCCAAGCTGAGGAAGAACTGCAAGGACCA

GACCCCCTACCTGACCTGCCTGAGGCTGGACAACAACAGCAGCCACATCGGCGTGTG

GCAGAAGAGGGCCGGCAGCAGCAGCAGCAGCCAGAACTGGGTGAAGCTGGTGGAGC

TGGGCGACGGCGTGAACGCCAGCGCCGGCGACACCGGCACCAACAAGCTGAAGAAG

GGCAACGACGACGTGGAGGAGGAGGACCAGAACAGCATGCAGATGATCGAGGAGCT

GCTGACCTGAGACATGCCCTGATGATGATGATGATGATGATGA

Supplementary Data S2

4.3. DNA extraction and polymerase chain reaction (PCR) amplification

The PCR volume was 25 μL, and each reaction contained 12.5 μL of 2 × Phanta Max Buffer, 0.5 μL of dNTPs, 0.5 μl of the forward primer (10 μM), 0.5 μL of the reverse primer (10 μM), 2 μL of template DNA, 8.5 μL of ddH2O, and 0.5 μL of Phanta DNA polymerase. After an incubation at 95°C for 5 min, 34 cycles of 95°C for 30 s, 55°C for 30 s, and 72°C for 45 s were performed, followed by a final incubation at 72°C for 5 min. PCR products were separated on a 1% agarose gel, excised, and extracted in 1× EDTA (TAE) buffer.

Supplementary Data S3

4.4. Preparation of the plant fusion expression vector and production of transgenic Arabidopsis

The 20 μL ligation reaction system contained the following components: 7 μL of the pcambia13021 expression vector, 2 μL of LR enzyme mixture, 4 μL of CE buffer (pH 8), 4 μL of the purified PCR product, and sterilized double distilled water up to a volume of 20 μL, and the mixture was incubated at 37°C for 30 min. The ligation product was transformed into E. coli DH5α using the heat shock method, and bacterial cells were evenly distributed on Luria-Bertani solid culture medium containing 100 mg·L-1 kana-mycin. After incubation of the inverted plates at 37°C overnight, a single colony was selected for PCR identification and sent to a biological company for sequencing, and the recombinant vector SHN3-P1302 was finally obtained. The constructed plant recombi-nant vector was transformed into Agrobacterium GV3101 and stored at -80°C until use. For homologous recombination, a Novartis kit was used (ClonExpress Multis One Step Cloning Kit). Standard methods were used for PCR, restriction enzyme digestion, plasmid DNA isolation and gel electrophoresis. The method for constructing P1302-BrSHIN3 was similar to that described previously [29]. Agrobacterium carrying the target gene was introduced into Arabidopsis using the flower dip method [30]. After the Arabidopsis siliques were mature, the seeds were collected, positive plants were screened on half MS solid medium containing, and the T1 genera-tion of transgenic plants grown on the selection medium were transplanted to the substrate. middle. Leaf DNA was extracted from transgenic and wild-type Arabidopsis plants. Wild-type Arabidopsis was

used as a control, and the specific primers BrSHINE3-F:GGACTCTTGACCATGGATGAACACTACTAAAGACCTTTCTCAAAAAG and mGFP-R: CTGACAGAAAATTTGTGCCC were used for positive PCR identifica-tion.

Supplementary Data S4

4.7. Gene expression analysis

Quantitative PCR (qPCR) was performed according to the instructions of the TransStart Tip Green qPCR SuperMix kit from Quanshijin Biotechnology Co., Ltd. The qPCR conditions were as follows: 94°C for 120 s, followed by 40 cycles of 94°C for 15 s, 65°C for 30 s, and 72°C for 30 s. The actin gene ID number of Chinese cabbage was Bch01G039600 (primer: BrACTIN1-F: GAATCCACGAGACGACTTACAAC BrACTIN1-R: CCTTAATCTTCATGCTGCTTGGT). For reference, 3 biological replicates and 3 technical replicates of each sample were analysed, and the relative expression levels were analysed using the $2^{-\Delta\Delta ct}$ method.
